# Empowering healthier lifestyles: patterns of digital tool use, self-efficacy, and affective balance in IBD-patients and a community sample

**DOI:** 10.3389/fdgth.2026.1710921

**Published:** 2026-06-30

**Authors:** Blanka Festő, Beatrix Rafael, Ábel Molnár, Tamás Szűcs, Sára Imola Csuka, Sanela Tóth-Njers, Anett Dávid, Barbara Horvát, Viola Sallay, Tamás Molnár, Tamás Martos

**Affiliations:** 1 Doctoral School of Clinical Medicine, University of Szeged, Szeged, Hungary; 2 Department of Preventive Medicine, Szegedi Tudomanyegyetem Szent-Gyorgyi Albert Orvostudomanyi Kar, Szeged, Hungary; 3 University of Szeged, Szeged, Hungary; 4 Department of Personality, Clinical and Health Psychology, Institute of Psychology, University of Szeged, Szeged, Hungary; 5 Department of Cognitive and Neuropsychology, Institute of Psychology, University of Szeged, Szeged, Hungary; 6 Albert Szent-Györgyi Medical School, Department of Internal Medicine, Center of Gastroenterology, University of Szeged, Szeged, Hungary; 7 Faculty of Psychotherapy Science, Sigmund Freud Private University, Paris, France

**Keywords:** affective balance, digital empowerment, health-related personal strivings, inflammatory bowel disease, internet empowerment, latent class analysis, person-oriented approach, self-efficacy

## Abstract

**Background:**

When reaching health-related personal strivings, consistent self-management is crucial, which can be supported by digital technology use and online resources. Prior research highlights the importance of self-efficacy, emotional balance, and internet and digital empowerment as distinct but partly related resources; however, little is known about how these resources interact with each other.

**Objective:**

The current study aimed to explore how internet and digital empowerment, self-efficacy, and affective balance interact to support health-related personal strivings among IBD patients and a community sample. We hypothesized that these factors would form different and meaningful patterns that are differentially associated with psychological factors.

**Methods:**

A total of 308 participants (151 from a community sample and 157 with IBD) completed questionnaires assessing health-related personal strivings, internet and digital empowerment, self-efficacy, emotional balance, life satisfaction, depression, anxiety, stress, and health behaviors. Latent Class Analysis (LCA) was used to identify subgroups. Group comparisons were conducted using ANCOVA, adjusted for demographic and health-related covariates.

**Results:**

We found four latent profiles: "Highly Empowered Achievers” (*n* = 55), "Moderately Empowered Strugglers” (*n* = 106), "Balanced but IDT Avoiders” (*n* = 67), and "Vulnerable and Disempowered group” (*n* = 80). Highly Empowered Achievers showed significantly higher life satisfaction (*F* = 13.25, *p* < .001, ω² = 0.092), lower perceived stress (*F* = 5.90, *p* < .001, ω² = 0.038), lower anxiety (*F* = 4.17, *p* = .006, ω² = 0.028), and greater health goal realization (*F* = 25.65, *p* < .001, ω² = 0.191) than other groups. Participants with IBD were more likely to belong to the Vulnerable and Disempowered group (OR = 2.21, 95% CI = 1.04–4.67, *p* = .039) and less likely to be in the Highly Empowered Achievers group (OR = 0.12, 95% CI = 0.04–0.34, *p* < .001).

**Conclusions:**

Internet and digital empowerment, self-efficacy, and affective balance formed four latent profiles associated with subjective health, psychological well-being, and goal attainment. Chronic disease management interventions could benefit from boosting digital competencies and emotional coping resources. Internet-based cognitive behavioral interventions may help address emotional balance and digital empowerment, though longitudinal studies are still needed to evaluate causal relationships.

## Introduction

Reaching personal goals, especially health-related personal strivings, has become a major challenge in the modern world. This difficulty affects not only the healthy population but also those suffering from chronic illnesses. However, digital tools offer numerous opportunities for users, such as tracking physical activity, monitoring calorie intake, joining supportive communities, or searching for health-related information, which can help them manage their lifestyle. In this article, using a relevant subsample drawn from a representative South-Hungarian survey and an inflammatory bowel disease (IBD) patients' sample, we aimed to explore meaningfully different patterns of health empowerment types (i.e., internet and digital empowerment), self-efficacy and affective balance in the context of health-related personal strivings, using a person-oriented approach.

### Background

Personal strivings are inner representations of behavior; they are typical goals of a person, usually being organized into a pattern (1). According to positive psychology, happiness and subjective well-being are related to the existence and achievement of adequate goals (2, 3). More recently, the importance of health-related personal strivings has also been recognized in health psychology, as they correlate with health-related behavior, general quality of life, and subjective state of health (4–6).

Inflammatory bowel disease is a multigenic, chronic disease without medical cure, characterized by the inflammation of intestinal mucosa (7). This frequently causes symptoms such as diarrhea, abdominal pain, weight loss, bloody stools, fatigue, and bloating (7). IBD is usually divided into two main clinical types, Crohn's disease (CD) and ulcerative colitis (UC); however, other clinical conditions also exist. Crohn's disease typically affects any region of the intestinal tract discontinuously with transmural inflammation and fissuring ulceration, while the inflammation in ulcerative colitis follows a continuous pattern specifically in the mucosa and submucosa of the colon (7). Although most patients can live a normal life, they need regular medical control not only to avoid flare-ups but also to prevent the progression of the disease (8).

The prevalence of IBD is particularly high in Western countries, including the United States, the United Kingdom, and Scandinavian countries (9, 10). A similar pattern began to emerge in Hungary with a prevalence of 0.55% already at the beginning of 2010 (11). To maintain intestinal homeostasis, a balance between the microbiome and the intestinal immune system is crucial. Therefore, any genetic or environmental factor that affects the barrier function of the epithelium can disrupt these balancing mechanisms (12).

Digital health solutions and online resources can help monitor daily life, set goals, and engage in new activities. This may be beneficial both for healthy individuals and those with chronic illnesses. For example, managing IBD symptoms requires strict consistency and self-management of patients, thus the examination of health-related personal strivings is particularly important in this population. In our previous study (13), we assessed health-related personal strivings of patients living with Crohn's disease. Participants had to specify one or more health-related personal strivings, then choose the currently most important one, and answer questions about it. Our results showed that 95% of the chosen health-related strivings are in connection with the most important risk factors of Crohn's disease, and overall could be divided into four categories: physical activity, stress management, sticking to a healthy diet and quitting smoking. Measuring the frequency of internet and digital technology (IDT) use, about half of our population used the internet and digital apps at least weekly to complete their goal, besides, approximately one-quarter of them operated regularly with smart wristlets or smartwatches (13).

Empowering mechanisms may play a key role in supporting the realization of health-related personal strivings. Empowerment is a multi-dimensional social process that promotes the feeling of control over one's own life and boosts the ability in people to act on important issues (14). In this study, we assigned specific types of empowerment that could promote reaching health-related personal strivings: internet and digital empowerment. Digital empowerment involves the skillful use of specific digital devices and applications, such as mobile health apps, wearable trackers, or smartwatches, which enable individuals to monitor and manage their health directly. In contrast, internet empowerment focuses on accessing and using online platforms, including health-related websites, forums, and social media groups, where individuals can acquire knowledge, share experiences, and seek support from others. Consequently, in the context of health-related personal strivings, internet and digital empowerment may play an important role for patients, enabling them to take steps toward managing their health. A 2013 study showed the importance of the internet and digital technology in laic medicine: 72% of internet users in the US searched for health information in the previous year (15). Moreover, in 2015, 66% of the US population showed interest in managing their health by using mobile apps (16).

Self-efficacy (SE) is a social-cognitive construct, an important motivational factor of disease management. It refers to a person's perception of their capability to perform tasks and achieve or maintain goals (17). SE can be an important predictor of the outcomes of chronic diseases, such as cancer (18, 19), multiple sclerosis (20, 21), and inflammatory bowel disease (22, 23). Successful self-management interventions in IBD patients could be more effective when supporting self-efficacy. As research has not found a cure for IBD yet, the focus of treatment is to avoid flare-ups and maintain remission as long as possible. Self-management—such as adhering to a specific diet, quitting smoking, consulting regularly with doctors and taking medication—and self-efficacy (the belief which helps patients performing these tasks) play important roles in the progression of the disease. Higher self-efficacy was associated with lower daily life and emotional impact, fewer systemic symptoms, and fewer daily coping strategies; in other words, higher self-efficacy helps in the successful management of IBD. This association is also observed in patients with clinically inactive disease (24). Although internet and digital empowerment and self-efficacy are conceptually related, they represent partially distinct psychological resources. Self-efficacy primarily refers to an individual's generalized or domain-specific belief in their capability to successfully perform behaviors and manage challenges, whereas internet and digital empowerment reflect perceived competence, autonomy, critical understanding, and active engagement in using online health information and digital tools. In the context of health-related personal strivings, self-efficacy may support persistence and behavioral regulation, while digital empowerment may facilitate access to information, self-management resources, and digital coping strategies.

Affective balance is the result of the consideration of positive and negative feelings experienced over a period; it explains emotional responses when evaluating quality of life (25). In the context of health-related personal strivings, affective balance may affect the motivation and consistency with which individuals pursue their personal strivings. Positive affective balance can enhance adherence to health-related routines, whereas negative affect may hinder progress by reducing motivation and increasing stress levels, which are critical factors in managing chronic conditions like IBD. Research on cardiovascular diseases (CVD) shows that positive affective balance is a protective factor against cardiovascular diseases, and self-efficacy in regulating negative feelings helps adapt to CVD (26, 27), while negative affect is associated with worse health status in patients (28). Moreover, anxiety and emotional regulation strategies have an adverse impact on health behaviors. Patients with positive affect and well-being pick up more healthy behaviors over time; however, negative affect and fewer psychological resources promote negative behavioral changes in the long term (29). In a longitudinal study with IBD patients, depression and relapses were positively correlated, whereas anxiety, helplessness, and significant changes in patients' lifestyle exerted their negative effects through depression (30). In our previous research, we examined positive and negative feelings connected to IBD patients' personal strivings. 71% of our population experienced at least average levels of positive feelings (curiosity, calm, interest, satisfaction), and half of them reported negative emotions (anger, frustration, stress, anxiety, shame, sadness) while striving for their personal goal (13).

### Goal of this study

In the present research, we aim to explore complex interactions between internet and digital empowerment and psychological characteristics (i.e., self-efficacy and positive emotional balance) in health-related personal strivings of IBD patients and in a community sample as a comparison. We assumed that these characteristics—namely, internet and digital empowerment, self-efficacy and affective balance—form a holistic, integrated system that supports the pursuit of personal strivings. Therefore, we applied a pattern-oriented or person-oriented approach, which provides a holistic view of individuals. This approach simultaneously considers multiple interdependent characteristics and investigates how these variables interact and form distinct types/subgroups as variations of a complex system (31). Given the conceptual overlap between empowerment and self-efficacy, the person-oriented approach was considered particularly suitable to examine whether distinct configurations emerge in which digital/internet empowerment and self-efficacy can be disentangled.

Although the person-oriented framework mostly serves exploratory purposes, we assumed that subgroups feeling more empowered and showing a higher level of affective balance and self-efficacy will exhibit a higher level of subjective health status and subjective well-being, and a lower level of depression and anxiety. Furthermore, we assumed that more empowered and self-efficient subgroups perceive themselves as more successful in achieving their health-related personal strivings compared to subgroups with less empowered and self-efficient members.

## Methods

### Recruitment

Data were collected from two separate samples. The first phase took place between August and September 2022, utilizing a South-Hungarian representative sample recruited by a professional survey firm. Participants provided informed consent and met the following inclusion criteria: (1) age 18 or older, (2) no current or recent (past year) psychiatric treatment, and (3) consent to participate. Individuals who did not meet these criteria were excluded. This phase yielded 704 respondents from Southern Hungary, of whom 151 were included in the final analysis based on complete data, thus the final sample could not remain representative due to the non-random exclusion criteria. No participants in this sample had IBD.

The second data collection occurred between November 2022 and January 2023 at the IBD Centre of the Internal Medicine Clinic in Szeged, Hungary, and was assisted by undergraduate psychology students. Participants were approached during routine check-ups and completed paper-based questionnaires after providing informed consent. Participation was voluntary. Inclusion criteria were: (1) age 18 or older, (2) a confirmed IBD diagnosis based on international criteria, and (3) consent to participate. Individuals under 18 or with current or recent (past year) psychiatric treatment were excluded. Of the 377 respondents, 157 provided sufficient, complete data for analysis. The same questionnaire was used in both data collections.

### Participants

The final sample consisted of 308 participants, comprising 151 from Study 1 and 157 from Study 2—the majority of the sample identified as women (53,9%). Based on the criteria mentioned above, individuals with at least one missing answer were excluded from the final statistical analysis. The average age was 41,7 years (SD = 10,95 years). All participants used the internet daily, primarily for work and entertainment, and nearly all owned a smartphone. See Table 1 for detailed sociodemographic characteristics. Given the potential heterogeneity of the sample with respect to the examined variables, no prior distinction was made between healthy and diseased participants. The analysis aimed to explore whether one or more special subgroups would emerge, which could not be anticipated before examination.

**Table 1:** Demographic characteristics of participants.

Characteristic	Response category	Number of participants
Gender	Female	*n* = 166 (53.9%)
	Male	*n* *=* 142 (46.1%)
Residence	City	*n* *=* 182 (59.1%)
	Village	*n* *=* 85 (27.6%)
	Rural area	*n* *=* 40 (13%)
Internet use		*n* *=* 308 (100%)
	For work	*n* *=* 250 (81.1%)
	For entertainment	*n* *=* 235 (77.3%)
Smartphone	Ownership	*n* *=* 304 (99.3%)
Relationship status	Married	*n* *=* 147 (47.5%)
	Live together	*n* *=* 69 (22.6%)
	In a relationship without living together	*n* *=* 14 (4.6%)
	Co-existence	*n* *=* 12 (3.9%)
	Single	*n* *=* 64 (21%)

### Measures

#### Health striving assessment

We employed an adapted version of the Personal Project Analysis [PPA (32)] to examine participants' health-related strivings. They were prompted with “The health-related strivings you are actively working towards” and asked to list their current health-related personal strivings (e.g., weight control, exercise, diet, quitting smoking, mental well-being, sleep). Participants then identified their most important goal and evaluated it in terms of emotional balance (including both positive and negative emotions), internet use, and digital technology engagement.

Positive and negative emotions were each measured with three items (33). For example, one item asked, “How often did you feel anger and frustration regarding your health?” (negative emotion). In contrast, another sample item asked, “How often did you feel interest and curiosity about your health?” (positive emotion). Responses were given on a 5-point Likert scale (1 = very rarely, 5 = very often). Both scales demonstrated good reliability, with positive emotions (Cronbach's α = 0.876; McDonald's ω = 0.878) and negative emotions (Cronbach's α = 0.903; McDonald's ω = 0.906) showing high internal consistency. By integrating both negative and positive emotions, we derived an “emotional balance” variable, where 1 represents predominantly negative emotions (bottom 33.33% of the distribution), 2 represents balanced emotions (middle 33.33%), and 3 represents predominantly positive emotions (top 33.33%).

Participants reported how often they used the internet to seek health-related information, choosing from the options: never, rarely, sometimes, frequently, or very frequently. Those who selected any option other than “never” were asked to rate their sense of empowerment from online information, using four items (e.g., “Thanks to online health information, I am more conscious about my health”). The scale showed excellent reliability (Cronbach's α = 0.961; McDonald's ω = 0.961). EFA with maximum likelihood extraction and oblimin rotation confirmed unidimensionality (one factor with eigenvalue > 1 explaining 86.15% of variance; loadings ranged from 0.918–0.940). By integrating the empowerment level participants' experience from obtaining health information online, we calculated a mean empowerment score, with higher values indicating greater perceived empowerment from online health information.

Participants were asked whether they used digital devices to monitor health-related data. Those who responded affirmatively rated their sense of empowerment using four statements (e.g., “Digital devices make me feel more in charge of my health”) on a 5-point Likert scale (1 = I disagree, 5 = I fully agree). The scale demonstrated excellent reliability (Cronbach's α = 0.975; McDonald's ω = 0.975). EFA with maximum likelihood extraction and oblimin rotation confirmed unidimensionality (one factor with eigenvalue > 1 explaining 90.77% of variance; loadings ranged from 0.942–0.967). By incorporating the empowerment level participants feel from using digital devices for health purposes, we computed a composite empowerment score by averaging the four items, with higher values indicating greater perceived empowerment.

Health-striving-related self-efficacy (34) was measured using four statements (e.g., “I am generally able to manage situations concerning my health”), rated on a 5-point Likert scale (1 = not at all true, 5 = absolutely true). The scale showed excellent reliability (Cronbach's α = 0.878; McDonald's ω = 0.879). EFA with maximum likelihood extraction and oblimin rotation confirmed unidimensionality (one factor with eigenvalue > 1 explaining 64.31% of variance; loadings ranged from 0.714–0.873).

Satisfaction with current health-related accomplishments was assessed using four items (e.g., “I am pleased with what I have been doing for my health recently”), rated on a 5-point Likert scale (1 = not at all true, 5 = absolutely true). The scale demonstrated excellent reliability (Cronbach's α = 0.949; McDonald's ω = 0.950). EFA with maximum likelihood extraction and oblimin rotation confirmed unidimensionality (one factor with eigenvalue > 1 explaining 82.63% of variance; loadings ranged from 0.891–0.936).

#### Satisfaction with life

Life satisfaction was measured using the validated Hungarian Satisfaction With Life Scale (H-SWLS (35), which includes five items (e.g., “In most aspects, my life is almost ideal”). Responses were given on a 5-point Likert scale (1 = I totally disagree, 5 = I fully agree). The scale demonstrated excellent reliability (Cronbach's α = 0.882; McDonald's ω = 0.890). EFA with maximum likelihood extraction and oblimin rotation confirmed unidimensionality (one factor with eigenvalue > 1 explaining 61.74% of variance; loadings ranged from 0.721–0.835).

#### Depression, anxiety, and stress

Depression, anxiety, and stress were measured using the Hungarian short version of the Depression Anxiety and Stress Scale [DASS (36, 37)], with three items per construct (total of 9 items). Example items include: “I had difficulty initiating anything” (depression), “I observed that I was trembling (e.g., my hands)” (anxiety), and “I responded with intense anger to everything” (stress). Responses were rated on a 4-point Likert scale (0 = not typical at all, 3 = highly or very often typical). Reliability was satisfactory across all scales: depression (Cronbach's α = 0.751; McDonald's ω = 0.782), anxiety (Cronbach's α = 0.788; McDonald's ω = 0.799), and stress (Cronbach's α = 0.832; McDonald's ω = 0.837).

#### Self-rated health and health behaviors

Participants rated their current health status on a 5-point Likert scale, where 1 indicated “very bad” and 5 indicated “excellent”.

For the assessment of health behaviors, we questioned participants on alcohol consumption, smoking, and physical activity. Participants were asked whether they had consumed alcohol in the past month and whether they currently smoked. They also reported the number of days per week they engaged in vigorous physical activity. Detailed information on health and risk behaviors is presented in Table 2.

**Table 2:** Descriptives of health and risk behaviors.

Characteristic	Response category	Number of participants
Alcohol consumption	Never	*n* = 98 (32.8%)
	Once a month or less	*n* = 105 (35.1%)
	2–4 times a month	*n* = 60 (20.1%)
	2–3 times a week	*n* = 30 (10%)
	4–6 times a week	*n* = 4 (1.3%)
	Every day	*n* = 2 (0.7%)
Smoking	Non-smoker	*n* = 221 (71.8%)
	Occasional smoker	*n* = 13 (4.2%)
	Smoker	*n* = 74 (24%)
Physical activity	0 days	*n* = 100 (32.5%)
	1 day	*n* = 27 (8.8%)
	2 days	*n* = 55 (17.9%)
	3 days	*n* = 47 (15.3%)
	4 days	*n* = 23 (7.5%)
	5 days	*n* = 26 (8.4%)
	6 days	*n* = 8 (2.6%)
	7 days	*n* = 22 (7.1%)

### Statistical analysis

We chose LCA over LPA (Latent Profile Analysis) primarily because our indicators were treated as ordinal variables. LCA is specifically designed to handle categorical and ordinal data, providing a more appropriate framework for identifying distinct patterns without the strict distributional assumptions of traditional profile analysis. We chose LCA over LPA (Latent Profile Analysis) as the data did not follow a normal distribution. Each model was evaluated based on the Bayesian Information Criterion (BIC), sample size-adjusted Bayesian Information Criterion (SABIC), Akaike Information Criterion (AIC), bootstrap likelihood ratio test (BLRT), and entropy. To address the assumption of local independence, we conducted a preliminary correlation analysis of the indicators; no high correlations (all r < 0.70) were found that would suggest significant local dependence issues. We used multinomial logistic regression to examine sociodemographic and general health status predictors of class membership and analysis of covariance to gain a detailed overview about of the differences between the four groups on dimensions of important psychological characteristics.

Variables used later in Latent Class Analysis, namely internet empowerment, digital empowerment, self-efficacy and affective balance, were recoded into three-level ordinal scales, using equal percentile-based cut points. This approach was chosen to reduce the impact of non-normality and sparse response distributions, improve model stability, and retain an ordinal interpretation of low, moderate, and high levels of each construct. We discuss the possible disadvantages of this procedure in the Limitations section.

## Results

### Identification of latent classes

We conducted a series of Latent Class Analysis (LCA) models ranging from 2 to 10 classes using the recoded variables of internet empowerment, digital empowerment, self-efficacy, and affective balance as input. The fit indices for the 2- to 10-class models are shown in Table 4. We compared the results of all nine models and decided on the final number of retained classes based on a complex consideration of several criteria. The low AIC values indicated that the 4-class or the 5-class model had the best fit. We preferred the 4-class model over the 5-class model, as a non-significant BLRT *p* value (*p* = 0.086) indicated that including a fifth class could not significantly improve the explanatory power of the solution. BLRT *p* was 0.029 for the 4-class model. Moreover, AIC and SABIC also suggested that the 4-class model has superior data fit, taking complexity into account. Entropy for the four-class solutions was 0.752, indicating moderate class separation. Higher entropy values generally reflect better classification accuracy, whereas lower values indicate greater classification uncertainty (38, 39). Entropy was therefore considered as an indicator of classification precision, but not as a decisive criterion for selecting the number of classes (40). Although the 3-class model showed the lowest BIC (2618), the 4-class model was not far off (BIC = 2,631), and other indicators suggested the 4-class model over the 3-class model. Moreover, although the 5-class solution showed higher entropy, and, thus, better separation, this additional class yielded less parsimonious solution according to the BLRT tests. In sum, the four-class solution was retained for further analysis, based on the combined consideration of fit indices, parsimony, theoretical interpretability, and the non-significant improvement of the five-class solution. The four embedded latent classes were named as: “Highly empowered achievers”, “Moderately empowered strugglers”, “Balanced but IDT Avoiders”, and “Vulnerable and disempowered” groups.

**Table 3:** *Post-hoc* test for class predictors.

	Group 1–2		95%CI		Group 3–2		95%CI		Group 4–2		95%CI	
Predictors	*p*	Odds ratio	Lower	Upper	*p*	Odds ratio	Lower	Upper	*p*	Odds ratio	Lower	Upper
Gender 2–1	0.312	1.51	0.68	3.36	0.067	0.52	0.26	1.05	0.488	0.77	0.43	1.40
Age	0.907	1.00	0.96	1.04	0.002	1.05	1.02	1.09	0.517	1.01	0.98	1.04
Educational attainment 2–1	0.246	1.92	0.64	5.78	0.310	1.60	0.65	3.97	0.397	0.71	0.32	1.57
Educational attainment 3–1	0.281	1.81	0.62	5.30	0.631	1.24	0.51	3.01	0.175	0.59	0.28	1.26
Place of residence 2–1	0.070	0.44	0.18	1.07	0.213	1.66	0.75	3.66	0.893	1.07	0.54	2.11
Place of residence 3–1	0.181	0.47	0.16	1.42	0.451	1.43	0.57	3.58	0.125	0.61	0.27	1.39
Health status 2–1	0.005	9.30	1.96	44.10	0.002	4.71	1.81	12.29	0.372	0.74	0.38	1.44
Health status 3–1	<.001	17.23	3.32	89.43	<.001	10.11	3.20	31.97	0.455	1.45	0.55	3.80
IBD 1–0	<.001	0.12	0.04	0.34	0.037	0.44	0.02	0.95	0.039	2.21	1.04	4.67

**Table 4:** Fit indices for 2 to 10 classes.

Number of classes	AIC	BIC	SABIC	Entropy	G²	G² p
2	2,560	2,624	2,570	0.729	161.0	<.001
3	2,521	2,618	2,536	0.754	104.0	<.001
4	2,500	2,631	2,520	0.752	64.7	0.029
5	2,502	2,666	2,526	0.823	48.1	0.086
6	2,509	2,707	2,539	0.778	37.5	0.085
7	2,518	2,749	2,553	0.811	28.4	0.057
8	2,530	2,795	2,570	0.817	22.4	0.008
9	2,539	2,838	2,584	0.829	13.9	<.001
10	2,551	2,883	2,601	0.828	7.8	NaN

The descriptive statistics and comparisons of the class rank means on the initial variables for the 4-class LCA model are shown in Table 4. The “Highly empowered achievers” group (*N* = 55) had the highest mean scores across all four criteria. The “Moderately empowered strugglers” group (*N* = 106) scored similarly high in internet and digital empowerment but achieved lower mean scores in self-efficacy and affective balance. In contrast, the “Balanced but IDT avoiders” group (*N* = 67) scored high in self-efficacy and affective balance, similar to “Highly empowered achievers”, while it scored lower on internet and digital empowerment. Vulnerable and disempowered group (*N* = 80) had the lowest mean scores in nearly every criterion, except for digital empowerment, where their mean score did not differ from that of Balanced but IDT avoiders (*W* = 0.074, *p* = 1.000).

#### Predictors of class membership

We conducted a multinomial logistic regression (MLR) to examine sociodemographic and general health status predictors of class membership. In MLR, we used Moderately empowered strugglers, the largest group (*N* *=* 106) with medium mean scores on all four dimensions, as the reference category. The predictors included gender, age, educational attainment, place of residence, health status, and IBD. For nominal and ordinal variables, we designated male gender, basic level education (8th grade and vocational training), living in the capital, moderate or worse subjective health, and no-IBD status, respectively. The pseudo-R-squared (*N*agelkerke) value was 0.231, indicating that the demographic variables explained 23.1% of the variance in group membership (*p* < 0.001). MLR results are presented in Table 5.

**Table 5:** Descriptive statistics of the four classes.

Class	Affective balance mean(SD)	Internet empowerment mean(SD)	Digital empowerment mean(SD)	Self-efficacy mean(SD)	Size
Highly empowered achievers	2.69 (0.62)	2.75 (0.52)	2.82 (0.48)	2.85 (0.52)	55
Moderately empowered strugglers	1.86 (0.77)	2.30 (0.54)	2.37 (0.65)	1.61 (0.49)	106
Balanced but IDT avoiders	2.48 (0.50)	1.33 (0.47)	1.28 (0.45)	2.52 (0.50)	67
Vulnerable and disempowered	1.26 (0.52)	1.06 (0.24)	1.29 (0.46)	1.3(0.62)	80

Below, we give all group membership probabilities with reference to Moderately empowered strugglers. Importantly, because the Highly Empowered Achievers class was the smallest subgroup (*n* = 55) in the solution, odds ratios involving this class should be interpreted with caution. The relatively wide confidence intervals provided below, indicate considerable uncertainty around the point estimates. Thus, we regard the respective findings as evidence of group-membership tendencies rather than precise population-level estimates.

When comparing Balanced but IDT avoiders to Moderately empowered strugglers, the gender comparison (female vs. male) was marginally significant (*p* = 0.067; OR = 0.52, 95% CI = 0.26–1.05). Age significantly predicts group membership (*p* = 0.002), though its effect is relatively weak (OR = 1.05, 95% CI = 1.02–1.09). Compared to poor health, having a medium health status predicted a higher probability of belonging to Highly Empowered Achievers (OR = 9.30, 95% CI = 1.96–44.10, *p* = 0.005). Having good health compared to poor health, predicted higher probability of belonging to Highly empowered achievers (OR = 17.23, 95% CI = 3.32–89.4, *p* < 0.001). Compared to poor health, medium health status increases the likelihood of belonging to Balanced but IDT avoiders (OR = 4.71, 95% CI = 1.81–12.29, *p* = 0.002), while good health status further increases this probability (OR = 10.11, 95% CI = 3.20–31.97, *p* < 0.001). Regarding IBD, individuals with IBD had a lower probability of belonging to Highly empowered achievers compared to non-IBD individuals (OR = 0.12, 95% CI = 0.04–0.34, *p* < 0.001). Vulnerable and disempowered group compared to Moderately empowered strugglers only differed in IBD, where having IBD increases the likelihood of belonging in Vulnerable and disempowered group (OR = 2.21, 95% CI = 1.04–4.67, *p* = 0.039).

#### Class comparisons: psychological characteristics

We conducted a series of analyses of covariance (ANCOVA) to gain a detailed overview of the differences between the four groups on the dimensions of important psychological characteristics (Table 3). We adjusted the associations for the same sociodemographic and health status variables as covariates as in MLR (i.e., gender, age, educational attainment, place of residence, health status, and IBD, the variables that were). Tukey's HSD test was used for *post hoc* analyses. To mitigate the risk of Type I error inflation due to multiple testing, we prioritized the interpretation of effect sizes (*ω*2 and Cohen's d) alongside *p*-values to ensure the practical significance of the differences between classes. However, *post-hoc* comparisons involving the Highly Empowered Achievers class should be interpreted cautiously because of its relatively small class size.

We observed a significant main effect of group membership for life satisfaction (*F* = 13.25, *p* < 0.001, ω² = 0.092). *post-hoc* tests revealed that Highly empowered achievers scored significantly higher than Moderately empowered strugglers, Balanced but IDT avoiders, and Vulnerable and disempowered group (*p* < 0.001 in all cases). Specifically, Highly empowered achievers had significantly higher scores than Moderately empowered strugglers (Cohen's d = 1.053), Balanced but IDT avoiders (Cohen's d = 0.828), and Vulnerable and disempowered group (Cohen's d = 1.205).

For perceived stress, we found a significant main effect of group membership (*F* = 5.90, *p* < 0.001, ω² = 0.038). *post-hoc* tests showed significant differences between Highly empowered achievers and Moderately empowered strugglers, Balanced but IDT avoiders, and Vulnerable and disempowered group (*p* = 0.002, *p* = 0.002, *p* = 0.003, respectively), with Highly empowered achievers scoring lower than all three groups. The effect sizes for these comparisons were large (Cohen's d = −0.691, −0.701, and −0.705, respectively).

With regard to anxiety, group membership also exhibited a significant main effect (*F* = 4.17, *p* = 0.006, ω² = 0.028). *post-hoc* comparisons revealed that Highly empowered achievers had significantly lower scores than Moderately empowered strugglers (*p* = 0.008, Cohen's d = −0.599) and Vulnerable and disempowered group (*p* = 0.011, Cohen's d = −0.625).

Regarding depressive symptoms, we found a significant main effect of group membership (*F* = 5.24, *p* = 0.002, ω² = 0.038). *post-hoc* testing identified significant differences between Highly empowered achievers and Vulnerable and disempowered group (*p* = 0.021), Moderately empowered strugglers and Balanced but IDT avoiders (*p* = 0.015), and Balanced but IDT avoiders and Vulnerable and disempowered group (*p* = 0.004). Specifically, Highly empowered achievers scored lower than Vulnerable and disempowered group (Cohen's d = −0.585), Moderately empowered strugglers scored higher than Balanced but IDT avoiders (Cohen's d = 0.508) and Balanced but IDT avoiders scored lower than Vulnerable and disempowered group (Cohen's d = −0.629).

For the implementation of health-related personal strivings through digital technology, the main effect of group membership was significant (*F* = 13.01, *p* < 0.001, ω² = 0.104). *post-hoc* tests revealed significant differences between Highly empowered achievers and Balanced but IDT avoiders (*p* = 0.007, Cohen's d = 0.616), Highly empowered achievers and Vulnerable and disempowered group (*p* = 0.002, Cohen's d = 0.742), Moderately empowered strugglers and Balanced but IDT avoiders (*p* < 0.001, Cohen's d = 0.675), and Moderately empowered strugglers and Vulnerable and disempowered group (*p* < 0.001, Cohen's d = 0.801).

Finally, we found a significant main effect of group membership for health-related personal striving realization (F = 25.65, *p* < 0.001, ω² = 0.191). *post-hoc* tests indicated significant differences between Highly empowered achievers and all other groups (*p* < 0.001 for all comparisons), with Cohen's d values of 0.992, 0.870, and 1.755, respectively. Additionally, Moderately empowered strugglers differed significantly from Vulnerable and disempowered group (*p* < 0.001, Cohen's d = 0.763), and Balanced but IDT avoiders differed significantly from Vulnerable and disempowered group (*p* < 0.001, Cohen's d = 0.885).

## Discussion

The purpose of this study was to explore complex interactions between internet and digital empowerment and psychological characteristics, factors that potentially support the pursuit of health-related personal strivings. We found four embedded latent classes that we named: Highly empowered achievers, Moderately empowered strugglers, Balanced but IDT avoiders, Vulnerable and disempowered group. It is important to note that the group labels were intended as concise, descriptive summaries of the patterns observed in the data. As they are based on non-diagnostic self-report measures, they should be interpreted as reflecting relative differences rather than fixed or pathologizing characteristics. Moreover, since we applied cross-sectional design, these group memberships' associations with other characteristics do not provide strong evidence to causal relationships.

In the present sample, Highly empowered achievers were found to show a favorable pattern with high empowerment, self-efficacy and emotional balance in their health-related strivings, primarily consisting of healthy community respondents with a predominance of women. This group experienced the lowest level of stress, anxiety and depressive symptoms and exhibited the highest scores in both subjective well-being and subjective health status. Members of this group dedicated the most time and energy toward pursuing their health-related personal strivings compared to the other three groups, and sought the most for the help of internet and digital technology. Additionally, they rarely smoked or consumed alcohol. Important to note, however, that this class was the smallest in our sample (*N* = 55), and confidence intervals of certain results indicated relatively large imprecision of the associations' estimates.

Moderately empowered strugglers were less empowered than Highly empowered achievers, however, their empowerment scores were relatively high compared to Balanced but IDT avoiders and Vulnerable and disempowered group. Their emotional balance and self-efficacy were the second lowest among the four groups, with significantly higher levels of stress, anxiety, and depressive symptoms than Highly empowered achievers. In terms of group composition, the number of IBD patients in this group was nearly twice as high as that of participants from the community sample. They spent less time and energy to pursuing their health-related personal strivings compared to Highly empowered achievers, but more than Group 4, which could be associated with a moderate sense of progress toward their health-related personal strivings. The gender ratio in this group was more balanced, although women were slightly more predominant.

Balanced but IDT avoiders were similarly well-balanced and self-efficient as Highly empowered achievers, although they rarely used the internet or digital technology to achieve their health-related personal strivings and felt less empowered than both Highly empowered achievers and Moderately empowered strugglers. They spent less time and energy to their health-related personal strivings; however, in contrast, their subjective health status was significantly better than that of Moderately empowered strugglers and Vulnerable and disempowered group, and their subjective well-being reached an average level. Two-third of the group consisted of a healthy community sample, and the gender ratio was relatively balanced. In terms of health-related habits, they smoked significantly less than Moderately empowered strugglers and Vulnerable and disempowered group. Their subjectively good health status could be partially explained by the fact that they are not chronic patients, which did not negatively impact their daily quality of life. Additionally, their health-related personal striving may not require the same level of consistency as those of individuals with inflammatory bowel disease, where adherence to health-related personal strivings may be more closely linked to their well-being.

The Vulnerable and disempowered group was the least empowered, self-sufficient, and emotionally balanced, dedicating the least time and energy to their health-related personal strivings. Moreover, this group had the lowest levels of subjective well-being and health status among all the groups. They experienced the highest levels of depression, stress, and anxiety compared to others (significantly more depressed than Highly empowered achievers and Balanced but IDT avoiders) and they showed minimal progress toward achieving their health-related personal strivings. The gender ratio was equal, although more than two-thirds of the group members were IBD patients.

These results provide preliminary support for our assumption that more empowered, efficient, and balanced groups exhibited a higher level of subjective health status and subjective well-being, and a lower level of depression and anxiety. Although levels of depression and anxiety remained subjectively low in all the four groups, these results indicate that Vulnerable and disempowered group and Moderately empowered strugglers significantly felt more depressed than Highly empowered achievers. Out of all the groups, Highly empowered achievers could be described with the highest levels of all health-related variables, empowerment and psychological characteristics. They spent the most time and energy on the progression of their health-related personal strivings, and in parallel experienced themselves more successful in achieving them. In contrast, the Vulnerable and disempowered group was a vulnerable subgroup of the sample, significantly differing in a negative way in most of the health-related variables. Moreover, the class solution also suggested that internet/digital empowerment and self-efficacy did not merely reflect a single undifferentiated resource dimension. For example, Moderately Empowered Strugglers showed relatively high internet and digital empowerment but comparatively low self-efficacy and affective balance, whereas Balanced but IDT Avoiders showed the opposite configuration, with relatively high self-efficacy and affective balance but low internet and digital empowerment. This dissociation supports the analytical usefulness of retaining these constructs as separate indicators in the LCA.

Although pattern-oriented studies are scarce in health-related personal strivings research, empowerment itself has already been investigated in the context of chronic conditions. In a qualitative study, Zare et al. (41) identified five dimensions of IBD patients' empowerment, including self-care, psychological coping with disease, social interaction skills, disease-specific health literacy and self-evaluation, which partly show similarities with our research. In self-care category, “ability to perform self-care behaviors” contain dieting, exercising, performing enjoyable activities, following-up the treatment, etc. These self-care behaviors could be described as health-related personal strivings, and spending time and energy pursuing them could be an empowering factor for chronically ill respondents. A positive attitude to life in the category of psychological coping means positive thinking in disease control. This factor can be partially observed in our research, as evidenced by the positive affective balance in our study. Therefore, we may assume that this positive affective balance can also empower respondents’ coping mechanisms. In social interaction skills, the ability to obtain information via the Internet and ask for support from knowledgeable people has an empowering effect, which is similar to internet empowerment in the current research. The fifth empowering factor of IBD patients is self-efficacy itself, the belief in one's ability to perform tasks in order to control the disease. According to Zare et al. (41), empowered patients not only possess potential abilities but also utilize them to take actions that control their disease.

Zare et al. (41) findings could partially explain the differences between Moderately empowered strugglers and Vulnerable and disempowered group: Moderately empowered strugglers may use the skills associated with experiences of internet and digital empowerment to perform slightly better in the progression of their health-related personal strivings, whilst Vulnerable and disempowered group may lack some psychological characteristics that could help them progress with their health-related personal strivings. Depressivity does not differ significantly between these two groups, which means that although depressivity could be associated with a lack of motivation, the internet and digital empowerment may be used by the group members to balance the effects of being unmotivated. Considering psychological characteristics, members of Highly empowered achievers and Balanced but IDT avoiders differ in internet and digital empowerment, indicating that feeling less empowered could be linked to less invested time and energy in health-related personal strivings.

Overall, it can be hypothesized that the psychological characteristics we examined, including internet empowerment, digital empowerment, self-efficacy, and positive emotional balance, can collectively support the achievement of health-related personal strivings. As a consequence, their combined high presence may contribute maintaining subjective well-being. Highly empowered achievers, characterized by high levels of all psychological characteristics, performed the best in terms of health goal achievement, subjective well-being, and health status. Despite relatively favorable self-efficacy and affective balance, lower internet/digital empowerment was associated with less reported progress toward health-related personal strivings in the Balanced but IDT avoiders subgroup. In contrast, Moderately empowered strugglers, which mostly consists of IBD patients, showed high internet and digital empowerment scores. Compared to Vulnerable and disempowered group, which scores low across all scales, Moderately empowered strugglers performed slightly better in health goal achievement and other health-related variables. These associations are consistent with previous findings suggesting that greater self-efficacy, more positive affective balance, and stronger empowerment resources are generally associated with better psychological adjustment, health-related behaviors, and chronic disease self-management (19, 20, 25, 31).

## Limitations

Our research has several limitations which have to be taken into account when interpreting results. Firstly, we only selected those from the IBD and community sample, who had at least one health-related personal striving, answered all the questions in connection with their currently most important striving, and used internet and digital technology in order to reach their health goal.

The second limitation concerns the recoding of continuous variables into three-level ordinal indicators prior to latent class analysis. Although this approach improved model stability and interpretability, it necessarily reduced variability and may have influenced class separation and profile formation. Some subtle differences between participants may therefore have been obscured. Consequently, the present four-class solution should be treated as a descriptive typology of broad response patterns rather than as evidence of exact representations of natural groups. Future studies should examine whether similar latent structures emerge when continuous indicators or alternative categorization procedures are used.

The third limitation is that the majority of the collected data relied on Likert-scale ratings, and there is only a limited amount of research in health-related personal strivings using pattern-oriented approach, which makes it difficult to compare our results with those of other studies. In a future investigation, it would be useful to examine the resources of IBD patients who belong to the most resourceful group, in order to compare their psychological characteristics with those of individuals in more vulnerable groups. These groups, which include resourceful IBD patients, could serve as the basis for planning interventions aimed at improving the quality of life for those in the more vulnerable groups. Furthermore, the multinomial logistic regression results should be interpreted with caution due to the relatively small size of the “Highly empowered” class (*n* = 55). This may increase the risk of overfitting and unstable odds ratios, especially with wide confidence intervals. Future studies using larger samples are needed to confirm the reproducibility and stability of this subgroup.

Although IBD status was included as a predictor of latent class membership, the present study did not examine potential interaction effects between health status (IBD vs. community sample) and latent profile membership on psychological outcomes. Consequently, comparisons between these subgroups remain exploratory, and the associations require validation in larger, more balanced studies with clinical and non-clinical samples. Moreover, the solution's entropy also indicates a moderate separation. Therefore, some degree of classification uncertainty should be assumed. Consequently, some participants may not have been assigned to their most representative latent class with complete certainty, which may have attenuated observed between-group differences and influenced regression estimates in the subsequent analyses. Therefore, class-specific findings and practical implications should be interpreted cautiously.

Another limitation is that due to the cross-sectional design, temporal or causal relationships between empowerment, self-efficacy, affective balance, and psychological outcomes cannot be determined. Although the community sample was originally recruited to represent the South-Hungarian population, the final sample in this analysis cannot be considered representative due to missing data and eligibility criteria. Therefore the findings cannot be generalized to the broader Hungarian and IBD populations without caution, particularly regarding digital engagement, motivation, or health awareness.

Finally, all major constructs were assessed using self-report measures. This may have increased shared method variance and introduced social desirability or response-style biases. Future studies should complement self-report data with behavioral indicators of digital-tool use, objective health-behavior measures, or longitudinal assessments of goal progress.

## Conclusions

The present study provides insight in the distinct person-level configurations of health-related personal strivings, especially with regard to internet and digital empowerment and their combination with self-efficacy and affective balance. Whereas the results are in line with previous studies, pointing out the positive association between self-efficacy, affective balance and digital/internet empowerment and health self-management, our study using a person-oriented approach extends these variable-centered findings that examined empowerment, self-efficacy, and affective balance separately (20, 22, 30, 31). While earlier studies mainly focused on linear associations between these variables and health-related outcomes, the present findings demonstrate that these resources may form distinct psychological configurations associated with different levels of well-being, stress, and health-related goal attainment. For example, the contrast between Moderately Empowered Strugglers and Balanced but IDT Avoiders illustrates that digital/internet empowerment and self-efficacy are different resources in everyday health-related striving.

Finally, the results' general patterns suggest that psychological resources in this study are worth to be supported through targeted interventions. To improve internet and digital empowerment, it could be useful to provide access to reliable websites and teach the use of internet and digital tools both for patients in chronic conditions and the wider public. To help promote positive emotional balance, techniques such as reframing and cognitive-behavioral therapy (CBT) could be beneficial. Self-efficacy can also be successfully developed through cognitive-behavioral therapy techniques, as well as the use of motivational interviewing (42, 43). ICBT (internet-based CBT) interventions however could allow professionals to provide double-focused care: it may help reduce even subclinical depressive symptoms thus help develop more effective coping strategies, which may contribute to an individual's increased well-being. On the other hand, it may enable individuals to learn using the internet as a resource, which could help users be more digitally empowered beyond being more affectively balanced. Furthermore, internet-based programs or mobile apps offer a cost-effective and rapid solution which could be widely spread between users, without the need for a huge number of trained therapists (44).

## Data Availability

The raw data supporting the conclusions of this article will be made available by the authors upon reasonable request. Requests to access the datasets should be directed to Sára Imola Csuka, csuka.sara@szte.hu.

## References

[1] Emmons RA. Personal strivings: an approach to personality and subjective well-being. J Pers Soc Psychol. (1986) 51(5):1058–68. 10.1037/0022-3514.51.5.1058

[2] Brunstein JC Maier GW Dargel A. Persönliche ziele und lebenspläne: subjektives wohlbefinden und proaktive entwicklung im lebenslauf. In: Brandtstädter J Lindenberger U, editors. Entwicklungspsychologie der Lebensspanne. Ein Lehrbuch. Stuttgart: Kohlhammer (2007). p. 270–304.

[3] Little BR Salmela-Aro K Phillips SD. Personal Project Pursuit: Goals, Action, and Human Flourishing. Mahwah, NJ: Lawrence Erlbaum Associates (2007). ISBN: 9780805858919.

[4] Boekaerts M Maes S Karoly P. Self-regulation across health behavior domains: concepts, theories and central issues. Appl Psychol. (2005) 54(2):149–54. 10.1111/j.1464-0597.2005.00201.x

[5] Boersma SN Maes S van Elderen T. Goal disturbance in relation to anxiety, depression, and coping in patients after coronary artery bypass graft surgery. J Health Psychol. (2005) 10(5):629–38.

[6] Schwarzer R. Self-regulatory processes in the adoption and maintenance of health behaviors. J Health Psychol. (1999) 4(2):115–27. 10.1177/135910539900400208 22021474

[7] Hanauer SB. Inflammatory bowel disease: epidemiology, pathogenesis, and therapeutic opportunities. Inflamm Bowel Dis. (2006) 12(1):S3–9. 10.1097/01.mib.0000195385.19268.68 16378007

[8] Podolsky DK. Inflammatory bowel disease New England. J Med. (2002) 347(6):417–29. 10.1056/NEJMra020831 12167685

[9] Andres PG Friedman LS. Epidemiology and the natural course of inflammatory bowel disease. Gastroenterol Clin North Am. (1999) 28(2):255–81. 10.1016/S0889-8553(05)70056-X 10372268

[10] Loftus EV. Clinical epidemiology of inflammatory bowel disease: incidence, prevalence, and environmental influences. Gastroenterology. (2003) 126(6):1504–17. 10.1053/jgastro2004.01.063 15168363

[11] Kürti Zs. Epidemiology, treatment and possible treatment complications of inflammatory bowel diseases according to a national survey. (Doctoral thesis). Hungarian: Doktori értekezés. Semmelweis Egyetem, Klinikai Orvostudományok Doktori Iskola, Budapest (2018). [A gyulladásos bélbetegségek előfordulása és kezelésének jellemzői országos felmérés alapján, valamint a kezelések lehetséges szövődményei]. . Available online at: http://old.semmelweis.hu/wp-content/phd/phd_live/vedes/export/kurtizsuzsanna.d.pdf (Accessed October 09, 2022).

[12] Nell S Suerbaum S Josenhans C. The impact of the microbiota on the pathogenesis of IBD: lessons from mouse infection models. Nat Rev Microbiol. (2010) 8(8):564–77. 10.1038/nrmicro2403 20622892

[13] Festő B Njers S Dávid A Horvát B Sallay V Molnár T. Health-related personal strivings amongst patients with crohn’s disease. The role of the internet and digital technology. Orv Hetil. (2023) 164(28):1102–10. 10.1556/650.2023.32801 37454328

[14] Page N Czuba CE. Empowerment: what is it? J Ext. (1999) 37(5). 10.66752/1077-5315.7834 (Accessed July 25, 2025)

[15] Fox S Duggan M. Health Online 2013. Washington: Pew Research Center (2013). Available online at: https://www.pewresearch.org/internet/2013/01/15/health-online-2013/ (Accessed August 17, 2025).

[16] Shah VN Garg SK. Managing diabetes in the digital age. Clin Diabetes Endocrinol. (2015) 1:16. 10.1186/s40842-015-0016-2 (Accessed May 20, 2025). 28702234 PMC5471958

[17] Bandura A. Self-efficacy: toward a unifying theory of behavioral change. Psychol Rev. (1977) 84(2):191–215. 10.1037/0033-295X.84.2.191 847061

[18] Yildiz B Korfage IJ Deliens L Preston NJ Miccinesi G Kodba-Ceh H. Self-efficacy of advanced cancer patients for participation in treatment-related decision-making in six European countries: the ACTION study. Support Care Cancer. (2023) 31(9):512. 10.1007/s00520-023-07974-2 37552324 PMC10409662

[19] Mystakidou K Tsilika E Parpa E Gogou P Theodorakis P Vlahos L. Self-efficacy beliefs and levels of anxiety in advanced cancer patients. Eur J Cancer Care. (2010) 19(2):205–11. 10.1111/j.1365-2354.2008.01039.x 19659666

[20] Riazi A Thompson AJ Hobart JC. Self-efficacy predicts self-reported health status in multiple sclerosis. Mult Scler. (2004) 10(1):61–6. 10.1191/1352458504ms986oa 14760954

[21] Schmitt MM Goverover Y Deluca J Chiaravalloti N. Self-efficacy as a predictor of self-reported physical, cognitive, and social functioning in multiple sclerosis. Rehabil Psychol. (2014) 59(1):27–34. 10.1037/a0035288 24320946 PMC4138971

[22] Keefer L Kiebles JL Taft TH. The role of self-efficacy in inflammatory bowel disease management: preliminary validation of a disease-specific measure. Inflamm Bowel Dis. (2011) 17(2):614–20. 10.1002/ibd.21314 20848516 PMC3005084

[23] Taft TH Keefer L Artz C Bratten J Jones MP. Perceptions of illness stigma in patients with inflammatory bowel disease and irritable bowel syndrome. Qual Life Res. (2013) 20(9):1391–9. 10.1007/s11136-011-9883-x 21424542

[24] Sheehan JL Greene-Higgs L Swanson L Higgins PDR Krein SL Waljee AK. Self-Efficacy and the impact of inflammatory bowel disease on Patients’ daily lives. Clin Transl Gastroenterol. (2023) 14(6):e00577. 10.14309/ctg.0000000000000577 36881812 PMC10299768

[25] Luque B Castillo-Mayén R Cuadrado E Gutiérrez-Domingo T Rubio SJ Arenas A. The role of emotional regulation and affective balance on health perception in cardiovascular disease patients according to sex differences. J Clin Med. (2020) 9(10):3165. 10.3390/jcm9103165 33007817 PMC7599936

[26] Boehm JK Kubzansky LD. The heart’s content: the association between positive psychological well-being and cardiovascular health. Psychol Bull. (2012) 138(4):655–91. 10.1037/a0027448 22506752

[27] Caprara GV Steca P. Self-efficacy beliefs as determinants of prosocial behavior conducive to life satisfaction across ages. J Soc Clin Psychol. (2005) 24(2):191–217. 10.1521/jscp.24.2.191.62271

[28] Chauvet-Gelinier JC Bonin B. Stress, anxiety and depression in heart disease patients: a major challenge for cardiac rehabilitation. Ann Cardiol Angeiol. (2017) 66(2):65–71. 10.1016/j.rehab.2016.09.002 27771272

[29] Van Kleef GA Côté S. The social effects of emotions. Annu Rev Psychol. (2022) 73:629–58. 10.1146/annurev-psych-020821-010855 34280326

[30] Mardini HE Kip KE Wilson JW. Crohn’s disease: a two-year prospective study of the relationship between psychological distress and disease activity inflammatory bowel diseases. Dig Dis Sci. (2004) 10(3):302–7. 10.1023/b:ddas.0000020509.23162.cc 15139504

[31] Sarradon-Eck A Bouchez T Auroy L Schuers M Darmon D. Attitudes of general practitioners toward prescription of mobile health apps: qualitative study. JMIR Mhealth Uhealth. (2021) 9(3):e21795. 10.2196/21795 33661123 PMC7974757

[32] Little BR. Personal projects and the distributed self: aspects of a conative psychology. In: Suls JM, editor. Psychological Perspectives on the Self. London: Lawrence Erlbaum Associates Publishers (2014). Vol. 4. p. 157–86.

[33] Martos T Sallay V Kézdy A. Everyday goals, religious motivations, and well-being: the mediating role of emotions. Stud Psychol. (2013) 55:221–7. 10.21909/sp.2013.03.638

[34] Rózsa S Réthelyi J Stauder A Susánszky É Mészáros E Skrabski Á. A hungarostudy 2002 országos reprezentatív felmérés általános módszertana és a felhasznált tesztbattéria pszichometriai jellemzői. Psychiatr Hung. (2003) 18:83–94.

[35] Martos T Sallay V Désfalvi J Szabó T Ittzés A. Psychometric characteristics of the Hungarian version of the satisfaction with life scale (SWLS-H). Mentálhigiéné és Pszichoszomatika. (2014) 15(3):289–303. 10.1556/mental.15.2014.3.9

[36] Parkitny L McAuley J. The depression anxiety stress scale (DASS). J Physiother. (2010) 56(3):204. 10.1016/S1836-9553(10)70030-8 20795931

[37] Szabó M. The short version of the depression anxiety stress scales (DASS-21): factor structure in a young adolescent sample. J Adolesc. (2010) 33(1):1–8. 10.1016/j.adolescence.2009.05.014 19560196

[38] Celeux G Soromenho G. An entropy criterion for assessing the number of clusters in a mixture model. J Classif. (1996) 13(2):195–212. 10.1007/BF01246098

[39] Nylund KL. Latent transition analysis: modeling extensions and an application to peer victimization. (Doctoral dissertation). University of California: Los Angeles (2007).

[40] Van Lissa CJ Garnier-Villarreal M Anadria D. Recommended practices in latent class analysis using the open-source R-package tidySEM. Struct Equ Model Multidiscip J. (2024) 31(3):526–34. 10.1080/10705511.2023.2250920

[41] Zare M Rezaei-Adaryani M Sharif F. Empowerment process in patients with inflammatory bowel disease: a grounded theory study. J Clin Nurs. (2020) 29(17–18):3343–53.

[42] Selçuk-Tosun A Zincir H. The effect of a transtheoretical model-based motivational interview on self-efficacy, metabolic control, and health behaviour in adults with type 2 diabetes mellitus: a randomized controlled trial. Int J Nurs Pract. (2019) 25(4):e12742. 10.1111/ijn.12742 31090161

[43] Yang M Viladrich C Cruz J. Examining the relationship between academic stress and motivation toward physical education within a semester: a two-wave study with Chinese secondary school students. Front Psychol. (2022) 13:965690. 10.3389/fpsyg.2022.965690 36186397 PMC9524391

[44] Zhou T Li X Pei Y Gao X. Internet-based cognitive behavioral therapy for subthreshold depression: a systematic review and meta-analysis. BMC Psychiatry. (2016) 16:356. 10.1186/s12888-016-1061-9 27769266 PMC5073460

